# Letter to the Editor: Inappropriate subcutaneous implantable cardioverter‐defibrillator discharge related to myopotential oversensing

**DOI:** 10.1002/joa3.12415

**Published:** 2020-08-11

**Authors:** Godefroy Chery, Larry Jackson, Kevin L. Thomas

**Affiliations:** ^1^ Department of Medicine Duke University Medical Center Durham NC USA; ^2^ Duke Clinical Research Institute Durham NC USA

*Dear Editor,*

It is reported that approximately 8% of patients with subcutaneous implantable cardioverter‐defibrillator (ICD) experience inappropriate ICD shocks.^1^ T‐wave oversensing (TWOS) remains the most common etiology of inappropriate ICD therapy in subcutaneous ICDs with a reported incidence of 60%–80%.^2^, ^3^, ^4^ However, other etiologies of inappropriate ICD therapy in subcutaneous ICDs often remain go unrecognized. Herein, we report a case of an inappropriate S‐ICD therapy secondary to myopotential oversensing following a change in QRS morphology coupled with a lower R‐wave amplitude and postural change status post transcatheter valve in valve tricuspid valve replacement (TVR).

Briefly, a 42‐year‐old female with a history of hypertrophic cardiomyopathy, permanent atrial fibrillation, severe tricuspid valve regurgitation treated with bioprosthetic TVR on June 6, 2013, who underwent subcutaneous implantable cardioverter defibrillator (S‐ICD) implantation on September 2016 for primary prevention of sudden cardiac death. The indication for S‐ICD was nonsustained ventricular tachycardia captured on Holter monitor and family history of SCD. Prior to her S‐ICD implantation, her electrocardiogram (August 2016) revealed atrial fibrillation and left posterior fascicular block. In April 2017, she suffered an inappropriate shock due to TWOS. Review of her prior device interrogation revealed she had a decrease in her R‐wave sensing from implant measuring approximately 0.8 mV on the secondary vector in 2016 compared with 0.2‐0.4 mV in 2017. Her device was then reprogrammed on 4 April 2017. Specifically, we changed her secondary vector to alternate vector, and kept the detection zones for conditional shock and shock at 200 and 230, respectively.

Over the next several months the patient developed progressive fatigue, dyspnea, lower extremity edema, weight gain and was found to have recurrent severe tricuspid regurgitation on transesophageal echocardiogram in July 2017. She subsequently underwent a valve in valve transcatheter aortic valve replacement on 23 August 2017. Following her valve surgery, we noted a new right bundle branch block (RBBB) on her electrocardiogram. She had an uneventful postprocedure course until March 2019 when she received an ICD shock while lying in bed on her left side. Device interrogation revealed one shock episode. Further investigation of the S‐ICD revealed a lower amplitude R wave (approximately 0.2‐0.4 mV S‐ECG signals on the Alternate Sensing vector) with the aforementioned postural change in the setting of her recent transcatheter TVR. Moreover, her EKG revealed a new RBBB that was not present as noted above.

To our knowledge, this is the first case report of inappropriate S‐ICD therapy due to myopotential oversensing in the context of a change in QRS morphology (new RBBB) and lower R‐wave amplitude following a transcatheter valve in valve TVR. Specifically, the change in RBBB morphology coupled with the lower R‐wave amplitude in the setting of the aforementioned postural change, led to myopotential oversensing, triggering inappropriate ICD therapy.

Herein, to resolve this issue, we re‐analyzed all sensing vectors to determine if alternative vectors would provide a better subcutaneous electrocardiogram (S‐ECG), and a new reference S‐ECG was captured at rest to optimize her signal. The new reference S‐ECG was evaluated for postural changes. The Alternate vector remained the optimal sensing configuration, and the device was reprogrammed from a 2‐Zone configuration (200 bpm conditional/230 bpm Shock only) to a 1 Zone configuration (230 bpm Shock only) on 20 March 2019, following the aforementioned changes she has not had further inappropriate shocks. We were able to optimize her device settings to prevent any additional inappropriate shock therapy. Consequently, an event (eg new structural or valvular disease, new myocardial infarction, newly detected conduction abnormalities, etc) with potential to result in morphology change, should warrant routine 12‐lead ECG to detect any change in QRS morphology and re‐evaluation of sensing channels and signals of S‐ICD to ensure proper optimization of settings.

## CONFLICT OF INTEREST

Authors declare no conflict of interests for this article.
